# Successful outpatient parenteral antibiotic therapy with cefiderocol for osteomyelitis caused by multi-drug resistant Gram-negative bacteria: a case report

**DOI:** 10.1093/jacamr/dlae015

**Published:** 2024-02-07

**Authors:** Paul Schellong, Janett Wennek-Klose, Christian Spiegel, Jürgen Rödel, Stefan Hagel

**Affiliations:** Institute for Infectious Diseases and Infection Control, Jena University Hospital—Friedrich Schiller University Jena, Am Klinikum 1, 07747 Jena, Germany; Hospital Care, Medipolis Intensive Care & Service GmbH Pharmaceutical OPAT Service Provider, Jena, Germany; Department of Trauma, Hand and Reconstructive Surgery, Jena University Hospital—Friedrich Schiller University Jena, Jena, Germany; Institute of Medical Microbiology, Jena University Hospital, Friedrich Schiller University, Jena, Germany; Institute for Infectious Diseases and Infection Control, Jena University Hospital—Friedrich Schiller University Jena, Am Klinikum 1, 07747 Jena, Germany

## Abstract

**Objectives:**

Post-traumatic osteomyelitis attributed to metallo-β-lactamase (MBL)-producing Gram-negative bacteria presents a challenging clinical scenario. Cefiderocol emerges as a viable treatment option within the limited therapeutic options available.

**Patient/case description:**

In this brief report, we present a case of a Ukrainian patient with osteomyelitis caused by multi-drug resistant *Pseudomonas aeruginosa*, which was successfully treated with cefiderocol, facilitated in part by outpatient parenteral antibiotic therapy (OPAT).

**Results and discussion:**

Administration of Cefiderocol via OPAT can present a safe and effective option for treatment of post-traumatic osteomyelitis with multi-drug resistant *Pseudomonas aeruginosa.* A possible effect on iron homeostasis of extended treatment duration with cefiderocol may be taken into consideration.

## Introduction

Recent reports have highlighted a significant prevalence of colonization and infection with multidrug-resistant bacterial strains in Ukrainian patients receiving treatment in European healthcare facilities.^1,2^ One particularly challenging clinical scenario involves post-traumatic osteomyelitis accompanied by foreign material, caused by metallo-β-lactamase (MBL)-producing Gram-negative bacteria.^3^ Cefiderocol, a parenteral siderophore-cephalosporin, has demonstrated both *in vitro* and clinical efficacy in infections caused by carbapenem-resistant Gram-negative bacteria, including MBL producers.^4–6^ In osteomyelitis, which requires prolonged treatment courses, outpatient parenteral antibiotic therapy (OPAT) can be considered as a possibility to reduce the duration of hospitalization and enhance patients’ quality of life.^7^ However, so far there is only limited experience with OPAT using cefiderocol.^8,9^ Previously, successful treatment of osteomyelitis with cefiderocol due to multi-drug resistant *Pseudomonas aeruginosa* and *Acinetobacter baumanii* have been reported in single cases.^9,10^ In this brief report, we highlight a successful case of OPAT using cefiderocol for the treatment of osteomyelitis caused by a MBL-producing Gram-negative bacterium.

## Patient/case description

In December 2022, a male Ukrainian soldier in his mid-thirties was presented with an infection-related pseudarthrosis of the left tibia to the department of trauma surgery of Jena University Hospital. In March of the same year, the injury took place amid the conflict between Russia and Ukraine, where he experienced a blast injury resulting in an open comminuted fracture of the left lower leg. Initially, he received early treatment from late April to mid-June 2022 at another hospital in Germany, which involved multiple surgical procedures, including a free flap tissue coverage and Ilisarov external fixator. Multiple bone cultures revealed the simultaneous presence of *Enterococcus faecalis* and NDM-producing *P. aeruginosa* (cefiderocol MIC 0.5 mg/L). During this external hospitalization, first ampicillin/sulbactam 3 g every 8 h were administered for 28 days, followed by cefiderocol 2 g every 6 h for 14 days.

When he presented at our hospital, we performed a debridement, sequestrectomy and repositioning of a comminuted tibia fracture using intramedullary nail osteosynthesis. Multiple cultures were taken from the tibial medullary cavity, which revealed the presence of NDM-producing *P. aeruginosa* (see Table 1). Definitive therapy was initiated with cefiderocol (2 g every 8 h). After 23 days of cefiderocol treatment, the patient was discharged and transitioned to OPAT via a PICC line for an additional 63 days, with no reported complications. Subsequently, another surgical intervention was performed, during which autogenous cancellous bone grafting impregnated with cefiderocol (4 g) was carried out. Therefore, bone marrow from the tibia was obtained with a Reamer-Irrigator-Aspirator-System, which then was dried and mixed with 4 g of cefiderocol powder and subsequently applied at the zone of bone defect. Microbiological examinations performed during this operation confirmed the ongoing presence of NDM-producing *P. aeruginosa* in the medullary cavity (cefiderocol MIC 0.38 mg/L, susceptibility testing performed with the same Etest as described in Table 1). Throughout a sequestrectomy of the tibial avascular bone and nail exchange were done. Systemic cefiderocol therapy was continued and therapeutic drug monitoring was performed to ensure adequate concentration. The trough concentration of cefiderocol was measured at 6.84 mg/L. One week later, another surgical revision was necessary due to an ulcerating postoperative wound infection. The intraoperative culture revealed *E. faecalis* and vancomycin-resistant *E. faecium*, but no longer *P. aeruginosa*. Consequently, linezolid was added to the treatment regimen.

**Table 1.** Antibiotic *in vitro* susceptibilities of the clinical isolate

Antibiotic	*Pseudomonas aeruginosa*	
	MIC (mg/L)	Interpretation^a^
Amikacin	≥64.0	R
Aztreonam	4.0	I
Aztreonam/avibactam	16/4	R
Cefiderocol	0.5	S
Ceftazidim	≥64.0	R
Ceftazidim/avibactam	>8/4	R
Ceftolozan/tazobactam	>8/4	R
Ciprofloxacin	≥4.0	R
Colistin	2	S
Gentamicin	—	—
Fosfomycin	—	—
Meropenem	≥16.0	R
Piperacilin/tazobactam	≥128.0	R
Tobramycin	≥16.0	R
Trimethoprim/sulfamethoxazole	—	—

Cefiderocol susceptibility testing was conducted with an Etest (Liofilchem^®^, Roseto degli Abruzzi, Italy) on Mueller–Hinton agar (ThermoFisher, Wesel, Germany).

R, resistant; I, susceptible with increased exposure; S, susceptible.

^a^According to EUCAST breakpoints, https://www.eucast.org/clinical_breakpoints.

**Figure 1.**
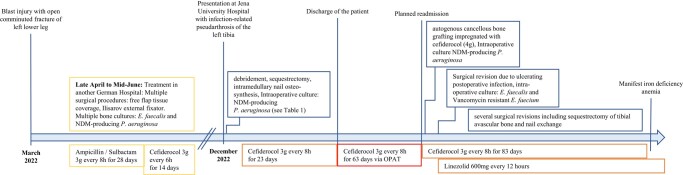
Timeline of the presented case.

Considering the patient's strong and explicit request to preserve the affected extremity, the antibiotic therapy was continued, and several surgical revisions were performed to address the ongoing infection. In total, the patient received 169 days of cefiderocol, with 63 days administered through OPAT. An overview of the treatment timeline of this case is provided in Figure 1.

Noteworthy, the patient showed iron deficiency anaemia, which was evident 10 days post-cefiderocol treatment cessation: haemoglobin 5.1 mmol/L (normal range 8.7–10.9 mmol/L, converted 8.22 g/dL), erythrocyte count 5.0 Tpt/L (4.5–5.9 Tpt/L), mean corpuscular volume 60 fL (80–96 fL), mean corpuscular haemoglobin 1.01 fmol (1.74–2.05 fmol), serum iron 1.5 µmol/L (6.3–30.1 µmol/L), transferrin saturation 2% (16%–45%), ferritin 11.4 µg/L (30–400 µg/L) and soluble transferrin receptor 25.9 mg/L (1.8–4.7 mg/L).

## Results and discussion

The OPAT administration of cefiderocol was carried out by a local experienced OPAT service. Stability data were used as provided in the Summary of Product characteristics. Briefly, diluted cefiderocol can be stored refrigerated up to 24 h and is stable at room temperature for up to 6 h. Owing to this limited stability, the OPAT service’s nurses daily prepared and delivered at noon three doses of cefiderocol (for noon, evening, following morning). To enable the recommended 3-hour infusion duration, a special elastomeric pump (EASYPUMP II ST 400-4-S with 300 mL of filling, B. Braun, Melsungen, Germany) was used. The patient had been trained in self-administration of the elastomeric pump by a nurse of the OPAT service. The patient was visited twice weekly by a nurse of the OPAT service also for dressing change and inspection of the PICC line. The patient was clinically monitored including laboratory measurements by his general practitioner once weekly.

No established trough concentration for cefiderocol exists for patients in this situation. Typically, a concentration exceeding four times the MIC is indicative of sufficient concentrations for β-lactam class antibiotics for bacterial killing. Hence, the measured total trough concentration of 6.84 mg/L was deemed adequate, reflecting a free plasma concentration of 7.5 times the MIC, considering the previously reported cefiderocol plasma binding of 58%.^11^ So far, only a few studies reported the results of cefiderocol therapeutic drug monitoring.^12,13^ Prinz *et al.* measured a median (interquartile range) trough concentration of 50.0 (27.2–74.6) mg/L in five critically ill patients. Gatti *et al.* reported trough plasma concentrations of the free fraction ranging from 0.59 to 56.78 mg/L in 13 patients. In summary, our observed trough concentration appears to be in the lower range, compared to previous reports, probably attributed to the normal kidney function in our patient.

Remarkably, despite the extended treatment duration, we did not observe the development of resistance to cefiderocol in *P. aeruginosa*, as previously reported.^14^ However, we observed a manifest iron deficiency anaemia at the end of the extended treatment duration, possibly related to the prolonged course of therapy with cefiderocol. Albeit, treatment emergent adverse effects related to iron homeostasis were very infrequently reported in patients receiving cefiderocol within the three Phase II/III trials and no evidence for a systemic impact on iron chemistry by the application of cefiderocol was observed during these trials.^5,15,16^ Additionally, a *post hoc* analysis of the APEKS-NP study, conducted recently, assessed the impact of cefiderocol administration on iron homeostasis and similarly concluded that cefiderocol treatment did not affect iron homeostasis.^17^ However, the treatment duration permitted in the study protocols ranged from 7 to 14 days only, with an extension allowed up to 21 days. In the case presented here, an iron deficiency anaemia developed after a postoperative anaemia during administration of cefiderocol. The prolonged treatment with cefiderocol might have hampered the iron uptake by a possible iron-scavenging effect after postoperative anaemia in addition to initially insufficient iron supply. An anaemia of chronic disease seemed to be unlikely due to the increased soluble transferrin receptor, low ferritin and low C-reactive protein (6.3 mg/L) at that time. We therefore propose to thoroughly investigate and treat anaemia and iron deficiency during prolonged courses of cefiderocol administration.
